# The impact of an exercise intervention on C - reactive protein during pregnancy: a randomized controlled trial

**DOI:** 10.1186/s12884-015-0576-2

**Published:** 2015-06-24

**Authors:** Marquis Hawkins, Barry Braun, Bess H. Marcus, Edward Stanek, Glenn Markenson, Lisa Chasan-Taber

**Affiliations:** Department of Biostatistics & Epidemiology, School of Public Health & Health Sciences, University of Massachusetts, Amherst, MA USA; Department of Kinesiology, School of Public Health & Health Sciences, University of Massachusetts, Amherst, MA USA; Department Family and Public Health, University of California San Diego School of Medicine, San Diego, CA USA; Baystate Medical Center, Springfield, MA USA

**Keywords:** C-reactive protein, Exercise, Pregnancy, Risk factors, Maternal health

## Abstract

**Background:**

C-reactive protein (CRP) during pregnancy has been associated with adverse maternal outcomes such as preeclampsia and gestational diabetes mellitus. Randomized trials suggest that exercise programs may be associated with reductions in CRP in non-pregnant populations; however, such studies have not been conducted among pregnant women. The purpose of this study was to evaluate the impact of an individually-tailored motivationally-matched exercise intervention on CRP in pregnant women.

**Methods:**

The Behaviors Affecting Baby and You study was a randomized controlled trial of prenatal physical activity to prevent the development of gestational diabetes mellitus in women at increased risk. Women were randomized to either a 12-week exercise intervention (*n* = 84) or a comparison health and wellness intervention (*n* = 87). High sensitivity CRP (mg/dL) was measured using a commercial immunoassay kit. Physical activity was measured using the Pregnancy Physical Activity Questionnaire. Mixed model analyses were used to evaluate the impact of the intervention on change in CRP using an intent-to-treat approach.

**Results:**

CRP decreased (−0.09 mg/dL, 95 % CI: −0.25, 0.07) from pre- to post-intervention in the exercise arm (*p* = 0.14) and increased (0.08 mg/dL, 95 % CI: −0.07, 0.24) (*p* = 0.64) in the health and wellness arm; however the between group difference was not statistically significant (*p* = 0.14). Findings did not differ according to ethnic group or pre-pregnancy body mass index. In a secondary analysis based on self-reported physical activity, women who decreased their time spent in sports/exercise experienced a mean increase in CRP (0.09 mg/dL, 95 % CI: −0.14, 0.33), whereas women who maintained or increased their sports/ exercise experienced a mean decrease in CRP (−0.08 mg/dL, 95 % CI: −0.23, 0.08) (*p* = 0.046).

**Conclusions:**

Findings from this randomized trial in an ethnically and socio-economically diverse population of pregnant women were consistent with a positive impact of the exercise intervention on CRP levels, but not of statistical significance.

**Clinical trial registration:**

ClinicalTrials.gov: NCT00728377. Registered 2 August 2008.

## Background

C-reactive protein (CRP) is an acute phase reactant protein often used as an indicator of chronic subclinical inflammation. In non-pregnant populations, elevated CRP has been associated with cardiovascular risk and the development of diabetes [1, 2]. Pregnancy is a pro-inflammatory state where markers such as CRP are elevated; however, this process is exaggerated in women that subsequently develop preeclampsia and gestational diabetes [3, 4]. For example, a review of 18 studies by Rebelo et al. that performed a meta-analysis on 7 found that CRP was higher in women who developed preeclampsia compared to women that experienced an uncomplicated pregnancy [5] and that this association seems to be modified by confounders, such as BMI. There is also evidence to suggest that the trajectory of CRP over the course of pregnancy is different in women who subsequently develop preeclampsia compared to women who have an uncomplicated pregnancy [6]. For example, a prospective cohort study by Teran et al. found that CRP levels at 32 weeks gestation and at delivery were higher in women that developed preeclampsia (*n* = 24) compared to women with a normal pregnancy (*n* = 183) despite similar CRP levels at 16 weeks gestation [6]. Further, CRP has been associated with oxidative stress and endothelial dysfunction [7], both of which are implicated in the development of preeclampsia [4]. Therefore, the identification of modifiable factors which can reduce or prevent increases in CRP levels during pregnancy could have important implications for the prevention of adverse maternal health outcomes.

Observational studies in non-pregnant populations have reported inverse associations between physical activity and CRP [8–12] with 6 % to 35 % lower CRP levels in physically active individuals compared to physically inactive individuals [12]. A review of 16 trials by Soares et al. examined the effect of lifestyle interventions (i.e. physical activity and/or diet) on inflammatory markers. Of the 10 studies that measured CRP, 2 reported no association and 8 reported reductions in CRP ranging from 30 % to 53 % with aerobic training or lifestyle (diet and exercise) interventions in non-pregnant women [13]. However, data on the association between physical activity and CRP levels during pregnancy is sparse [14–16]. In a recent observational study, 30 min increases in time spent in light intensity physical activity in the second trimester of pregnancy were associated with 0.4 mg/L lower levels of CRP among 294 participants in the NHANES study 2003–2006 cycles [14]. However, due to the cross-sectional nature of this study, the impact of physical activity on change in CRP could not be determined.

The association between physical activity and CRP is concerning given the relatively low levels of physical activity among pregnant women. Physical activity levels decline through pregnancy, even in women who are physically active prior to pregnancy [17, 18]. Compared to non-pregnant women, pregnant women are nearly half as likely to meet physical activity guidelines [19]. Among Hispanic women, physical activity levels are even lower. Data from the Behavioral Risk Factor Surveillance Systems showed that Hispanic women had a nearly 40 % lower odds of meeting recommended levels of physical activity compared to non-Hispanic white women [20].

To our knowledge, no studies have examined the impact of an exercise intervention on CRP during pregnancy. Thus, we evaluated the impact of individually-tailored motivationally-matched exercise intervention on CRP levels in an ethnically diverse sample of pregnant women at high risk for gestational diabetes mellitus (GDM).

## Methods

The Behaviors Affecting Baby and You (B.A.B.Y.) study was a randomized controlled trial of an exercise intervention with the overall goal of preventing the development of GDM in pregnant women at high risk. The aim of the current manuscript was to evaluate one of the secondary aims of the B.A.B.Y. study: the impact of the exercise intervention on change in CRP. The study was based in the ambulatory obstetrical practices of Baystate Medical Center, a large tertiary care facility in Western Massachusetts, from 2007 to 2012. Baystate Medical Center, serves an ethnically and socio-economically diverse population with approximately 4300 deliveries per year.

Health educators pre-screened eligible patients from 2007 to 2012 using demographic and medical characteristics provided on a daily roster of scheduled patients to generate a list of potential participants. Potential participants were asked if they would like to participate in the study and invited to participate; those who agreed were further screened for eligibility. Eligible women were in their first trimester of pregnancy, physically inactive (i.e., accumulated ≤30 min of moderate or vigorous intensity exercise on fewer than 3 days per week) and at high risk for GDM defined as either: 1) overweight/obese with a family history of diabetes or 2) a diagnosis of GDM in a prior pregnancy defined according to the American Diabetes Association criteria [21]. Exclusion criteria were: 1) <16 or >40 years of age, 2) history of type 2 diabetes, hypertension, heart disease, chronic renal disease, 3) current medications which adversely influence glucose tolerance, 4) contraindications to participating in moderate physical activity, 5) inability to read English at a 6^th^ grade level, or 6) nonsingleton pregnancy. All women enrolled signed a written informed consent. The Institution Review Board of the University of Massachusetts-Amherst and Baystate Medical Center approved this study.

### Study design

A detailed description of the study design has been presented elsewhere [22]. In brief, eligible women were recruited at a prenatal visit in early pregnancy (mean = 11 weeks gestation) and randomized to either a 12-week individually-tailored exercise intervention or a comparison health and wellness intervention. Randomization was stratified based on age (<30, >30 years), prepregnancy body mass index (BMI) (overweight: >25 kg/m^2^ vs. normal weight BMI <25 kg/m^2^), and ethnicity (Hispanic vs. non-Hispanic). Within each stratum, a blocked randomization was used such that both treatment groups are assigned an equal number of times in each set of four sequentially enrolled subjects. Women were not blinded to their assigned intervention group.

The intervention was kicked-off with an initial session with a health educator who administered a Tailoring Questionnaire, which assessed current stage of motivational readiness for physical activity adoption, and set behavioral goals. Over the course of the study, both intervention arms received monthly booster telephone calls at 2, 6, and 10 weeks from health educators that provided individualized feedback as well as reviewed participants’ progress toward their behavioral goals. Additionally, tip sheets were mailed weekly for the first 4 weeks of the intervention and then every other week thereafter. All intervention materials were written at a 6^th^ grade reading level.

### Theoretical model

The intervention drew from the Transtheoretical model [23] and social cognitive theory [24] constructs for physical activity which accounts for the individual’s stage of motivational readiness for change as well as the processes that help facilitate that change. It takes into account findings by our research group on the specific social, cultural, economic, and environmental resources as well as challenges faced by women of diverse backgrounds [25].

### Exercise intervention

The overall goal of the exercise intervention was to encourage pregnant women to achieve ACOG guidelines for physical activity during pregnancy (≥30 min of moderate intensity activity on most days of the week) [26]. The specific activities women engaged in were self-selected and included activities such as dancing, walking, and yard work. The weekly goals were to increase time spent in moderate intensity physical activity by 10 % each week to safely progress towards the overall activity goals. The participants were provided a digital pedometer and an activity diary to encourage self-monitoring.

The 65-item Tailoring Questionnaire assessed the participant’s current stage of motivational readiness for physical activity adoption, self-efficacy, decisional balance, use of cognitive and behavioral processes of change, and time spent in physical activity. In light of responses to this questionnaire, health educators discussed barriers and facilitators to adopting physical activity. A stage-matched manual targeting the specific stage of motivational readiness to adopt physical activity was then given to the participants. These manuals included the benefits of physical activity, tips for stretching, building social support, goal setting, and strategies for overcoming barriers to physical activity.

Participants’ progress toward their behavioral goals were assessed via a follow-up Tailoring Questionnaires and were mailed monthly with a postage paid return envelope. Based upon responses to these questionnaires, individually tailored reports were generated and mailed monthly to the participant along with the corresponding stage-matched manual. Each tailored report described the individual’s current stage of motivational readiness for becoming active, assessment of mediators for physical activity (i.e. self-efficacy, benefits and barriers for physical activity, cognitive and behavioral processes), normative feedback, and feedback regarding progress towards physical activity goals since prior assessment. If a tailoring questionnaire was not returned, staff contacted the participant to request that they send it. If the questionnaire could not be returned, the subsequent stage matched manual was based on responses to the previous returned questionnaire. Monthly booster telephone calls provided individualized feedback based on their motivational readiness for physical activity adoption.

### Health and wellness intervention

The health and wellness intervention received tip sheets and telephone booster calls on the same contact schedule as the exercise arm, which controlled for contact time, while keeping the content of the two interventions distinct. Specifically, after completion of the initial tailoring questionnaire, the health educator focused on general issues related to health and wellness during pregnancy instead of issues related to physical activity. A series of ACOG informational booklets on general issues related to health and wellness during pregnancy were mailed to the participants weekly during the first four weeks and then biweekly thereafter. These booklets were selected to represent high-quality standard low-cost self-help material currently available to the public. A follow-up tailoring questionnaire was mailed on week twelve. Monthly booster telephone calls provided individualized feedback on progress toward their health and wellness behavioral goals.

### Physical activity assessment

The Pregnancy Physical Activity Questionnaire (PPAQ) was used to measure physical activity prior to randomization (baseline) and at the end of the 12-week intervention period by interviewers blinded to the study arm. The PPAQ is a semi-quantitative instrument that has been previously validated in this study population [27]. The PPAQ queries the usual time spent participating in 32 activities of either light, moderate, or vigorous intensity during the past month in four activity types: household/caregiving, occupational, sports/exercise, and transportation. The number of minutes spent in each reported activity was multiplied by its metabolic equivalent of task (MET) level and summed to arrive at an estimate of average weekly MET-hours/week. MET intensity scores were based on the Compendium of Physical Activities [28], with the exception of walking and light housework activities, for which field-based measures among pregnant women were used [27]. In addition to total MET hours/week, physical activity was classified by intensity and type. We combined moderate and vigorous intensity physical activity into a category of moderate-vigorous activity because few women reported spending time in vigorous activity. To estimate hours per week of sedentary behavior, participants were asked to report the amount of time spent watching TV/videos or sitting/standing at home, work, or during transportation. We classified women as decreasing time spent in physical activity if they had >1 MET-hour/week decrease from baseline to post-intervention. Women were classified as maintaining/increasing time spent in physical activity if they maintained their activity (allowing for a ≤ 1 MET-hour/week decrease) or increased their activity from baseline to post-intervention.

### C - reactive protein (CRP)

High sensitivity CRP (mg/dL) was measured using a commercial immunoassay kit from Diagnostic Systems Laboratories Inc. Fasting serum samples were collected prior to randomization (baseline) and at the time of routine screening for GDM (24–28 weeks gestation) at Baystate Reference Laboratory. Serum samples were stored at −80° Celsius in a freezer at Baystate Reference Laboratory and shipped to the Energy Metabolism Laboratory at UMASS Amherst where analyses were performed. Day-to-day coefficient of variation for CRP was 7.5 %.

### Covariates

Descriptive characteristics including age, ethnicity, education, annual household income, marital status, living situation (e.g., with a spouse or partner), smoking status (pre-pregnancy and during pregnancy), and the number of adults and children in the household were collected at the time of enrollment via standardized questionnaires. Pre-pregnancy body mass index (BMI), gestational age, and gestational weight gain were abstracted from medical records. Gestational weight gain was calculated as change from pre-pregnancy weight to weight at delivery. Obesity was defined as pre-pregnancy BMI ≥30 kg/m^2^.

### Statistical analysis

Chi square tests and Fisher’s exact tests were used to compare the distribution of socio-demographic and descriptive characteristics between the intervention groups at baseline. A Wilcoxon Rank Sum test was used to compare median CRP levels between the intervention arms at baseline and post-intervention. The main study analyses were carried out using an intent-to-treat approach. CRP was log transformed (log-CRP) to correct for skewness. Mixed model analyses, using PROC mixed in SAS, were used to compare change in log-CRP between the exercise and the health and wellness arms. The mixed model used fixed treatment and assessment period effects. The random effects in the model corresponded to participants and days (nested in participants). A period by group interaction was used to compare differences in change in log-CRP in the exercise arm relative to the health and wellness arm. To determine if the impact of the intervention differed by important log-CRP risk factors, second order interactions between pre-pregnancy BMI (i.e. overweight, obese) and ethnicity (i.e. Hispanic, non-Hispanic), and change in log-CRP by intervention arm were then evaluated and retained in the model if significant at *p*<0.10. Finally, we performed a sensitivity analysis excluding women who were not compliant with the exercise intervention. Non-compliance was defined as a self-reported decrease in time spent in sports/exercise activities of moderate intensity or greater from baseline to post-intervention.

As a secondary aim, we examined the impact of self-reported change in physical activity on change in CRP from pre to post intervention. We created categories of change in time spent in sports/exercise activities of moderate intensity or greater; 1) decrease or 2) maintained/increased. A Wilcoxon Rank Sum test was used to compare median change in CRP between categories of change in physical activity.

Power calculations were based upon our sample size of 171 participants with a ratio of exposed to unexposed of 1:1. We had 80 % power to detect a mean difference in CRP between arms of 0.76 mg/dL based on a standard deviation of 1.50 mg/dL using a two group t-test with a 0.05 two-sided significance level.

## Results

**Fig. 1:**
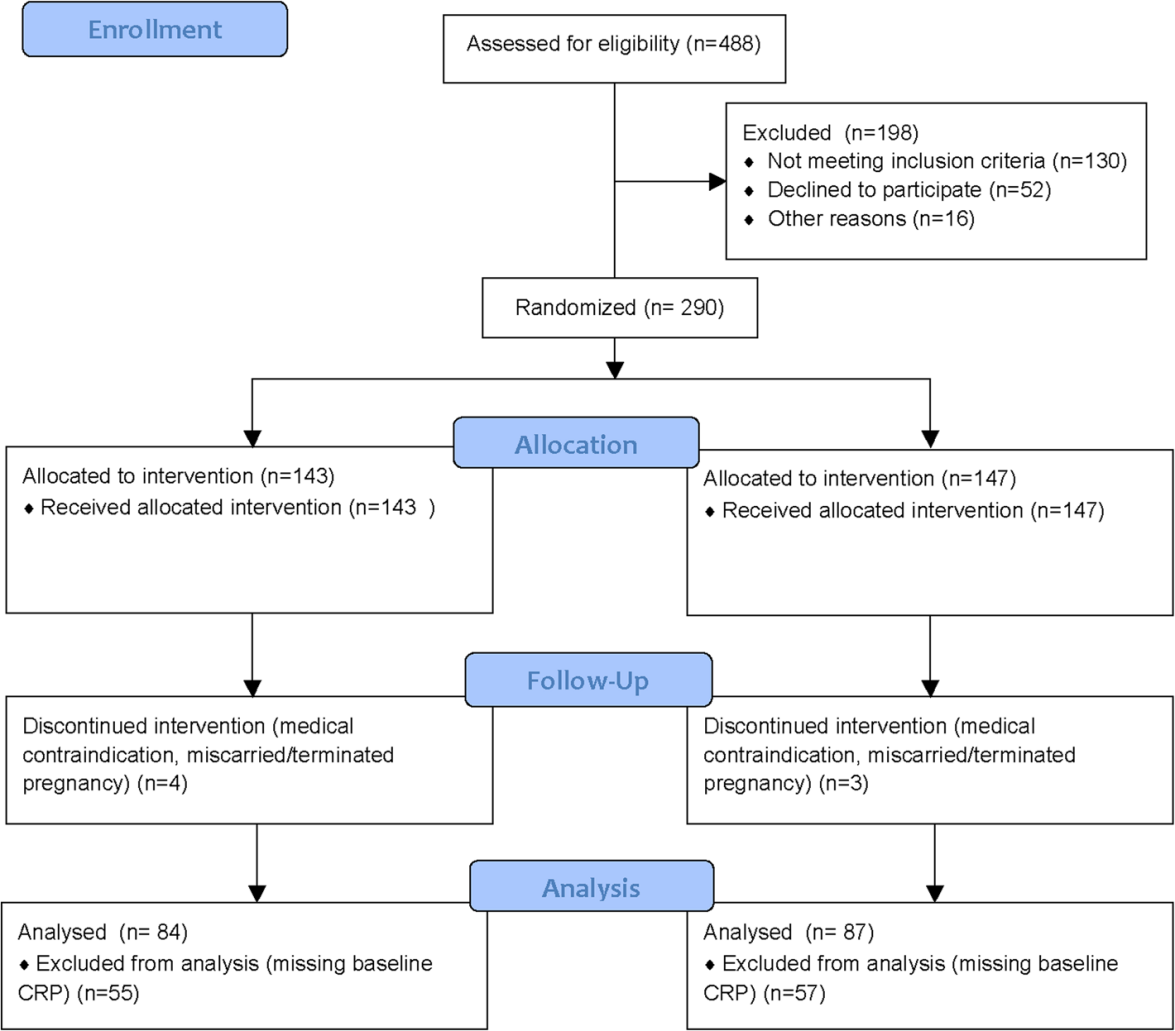
Flow diagram: The Behaviors Affecting Baby and You (B.A.B.Y.) Study; 2007–2012

The B.A.B.Y. study comprised a total of 290 women who were randomized into the exercise (*N* = 143) or the health and wellness (*N* = 147) intervention arms. Of this group, 178 women agreed to have their blood drawn at baseline and therefore had CRP data for analysis. Of this group, 7 were subsequently excluded due to the development of a medical contraindication or miscarriage/pregnancy termination. Therefore, the final sample for analysis included 171 participants; 84 in the exercise arm and 87 in the health and wellness arm (Fig. 1). There were no adverse effects of the intervention reported in either arm.

There were no statistically significant differences in physical activity, sedentary behavior, or any socio-demographic or medical history characteristic at baseline between women with or without CRP data, with the exception of obesity. Specifically, a higher proportion of women missing CRP data were obese compared to the women not missing CRP data (73 % vs. 55 %, respectively; *p* = 0.002).

Overall, the majority of women were young (mean age 26.5), Hispanic (57.3 %), low income (54.1 %; <$30,000 annual household income), unmarried (70.8 %), lived with a partner (60.8 %), had two or more adults living in the household (75.4 %), had at least one child in the household (71.9 %), and obese (55 %). Nearly all the women had a family history of diabetes (91.8 %), and 10.5 % had a prior diagnosis of GDM. There were no statistically significant differences between the intervention arms in any socio-demographic, medical history, or behavioral variables at baseline with the exception of income, and the number of adults and children in the household (Table 1). Specifically, a higher percentage of women in the exercise arm reported an annual household income <$30,000 (57 % vs. 50 %), had two or more adults in the household (86 % vs. 66 %), and had at least one child in the household (69 % vs. 53 %) compared to women in the health & wellness arm.

**Table 1:** Baseline characteristics of participants (N = 171); The B.A.B.Y. Study, 2007-2012

	Total population		Exercise		Health & wellness		*p*-value
	(*N* = 171)		(*N* = 84)		(*N* = 87)		
	N	%	N	%	N	%	
Sociodemographic Variables							
Age (years)							
16 to 19	24	14.0	14	16.7	10	11.5	0.49
20 to 24	63	36.8	33	39.3	30	34.5	
25 to 29	38	22.2	15	17.9	23	26.4	
≥30	46	26.9	22	26.2	24	27.6	
Ethnicity							
Hispanic	98	57.3	47	56.0	51	58.6	0.84
Non-Hispanic	73	42.7	37	44.0	36	41.4	
Education							
<High School	38	22.2	22	26.2	16	18.4	0.17
High School Graduate	52	30.4	24	28.6	28	32.2	
>High School	68	39.8	35	41.7	33	37.9	
Missing	13	7.6	3	3.6	10	11.5	
Income							
<$15,000	68	40.0	32	38.6	36	41.4	0.04
>$15,000-$30,000	24	14.1	16	19.3	8	9.2	
>$30,000	37	21.8	14	16.9	23	26.4	
Don’t know/refused/missing	41	24.1	21	25.3	20	23.0	
Marital Status							
Single	121	70.8	59	70.2	62	71.3	0.52
Married	48	28.1	25	29.8	23	26.4	
Refused/missing	2	1.2	0	0.0	2	2.3	
Living With Spouse/Partner							
Yes	104	60.8	55	65.5	49	56.3	0.18
No	55	32.2	26	31.0	29	33.3	
Missing	12	7.0	3	3.6	9	10.3	
Adults in Household (≥18 yrs)^a^							
1	33	19.3	11	13.1	22	25.3	0.01
2	85	49.7	46	54.8	39	44.8	
≥3	44	25.7	26	31.0	18	20.7	
Missing	9	5.3	1	1.2	8	9.2	
Children in Household (<18 yrs)^a^							
0	42	24.6	26	31.0	16	18.4	0.04
1	62	36.3	32	38.1	30	34.5	
2	44	25.7	20	23.8	24	27.6	
≥3	17	9.9	6	7.1	11	12.6	
Missing	6	3.5	0	0.0	6	6.9	
Medical History Variables							
Prepregnancy BMI							
<30 kg/m^2^	77	45.0	35	41.7	42	48.3	0.39
>30 kg/m^2^	94	55.0	49	58.3	45	51.7	
Previous GDM							
Yes	18	10.5	7	8.3	11	12.6	0.3
No	136	79.5	66	78.6	70	80.5	
Missing	17	9.9	11	13.1	6	6.9	
Family History of Diabetes							
Yes	157	91.8	76	90.5	81	93.1	0.52
No	3	1.8	1	1.2	2	2.3	
Missing	11	6.4	7	8.3	4	4.6	
Behavioral Variables							
Prepregnancy Smoking Status							
None	132	77.2	65	77.4	67	77.0	0.95
<10 cigarettes/day	17	9.9	9	10.7	8	9.2	
>10 cigarettes/day	2	1.2	1	1.2	1	1.1	
Missing	20	11.7	9	10.7	11	12.6	
Current Smoking Status							
None	110	64.3	53	63.1	57	65.5	0.18
<10 cigarettes/day	39	22.8	21	25.0	18	20.7	
>10 cigarettes/day	10	5.8	7	8.3	3	3.4	
Missing	12	7.0	3	3.6	9	10.3	

^a^Including the participants as appropriate: if <18 yrs, included as a child; if > 18 yrs, included as an adult

At baseline, there were no differences in CRP between interventions arms (Table 2). The geometric mean (95 % C.I.) CRP in the exercise arm was 0.83 (0.71, 0.98) mg/dL as compared to 0.68 (0.57, 0.82) mg/dL in the health and wellness arm (*p* = 0.21) (Table 2). After the 12-week intervention, there was a small and non-significant difference in change in CRP between the intervention arms (*p* = 0.14) (Table 2). Specifically, CRP decreased (−0.09 mg/dL, 95 % CI: −0.25, 0.07) from baseline to post-intervention in the exercise arm (*p* = 0.14), whereas there was a non-significant increase in CRP in the health and wellness arm (0.08 mg/dL, 95 % CI: −0.07, 0.24) (*p* = 0.64).

**Table 2:** Distribution of C-reactive protein at pre- and post-intervention by intervention arm: The B.A.B.Y. Study, 2007-2012

	Baseline				Post-intervention				Change			
	Geometric mean	95%	CI	p-value	Geometric mean	95%	CI	p-value	Mean (mg/dL)	95%	CI	p-value
Total Population												
Exercise Arm	0.83	0.71	0.98	0.21	0.75	0.60	0.94	0.86	−0.09	−0.25	0.07	0.14
Health and Wellness Arm	0.68	0.57	0.82		0.69	0.51	0.92		0.08	−0.07	0.24	
By Ethnicity												
Non-Hispanic												
Exercise Arm	0.83	0.65	1.10	0.20	0.60	0.41	0.88	0.84	−0.26	−0.55	0.04	0.08
Health and Wellness Arm	0.63	0.47	0.84		0.60	0.42	0.85		−0.07	−0.24	0.11	
Hispanic												
Exercise Arm	0.84	0.66	1.10	0.57	0.82	0.59	1.14	0.54	−0.04	−0.23	0.14	0.42
Health and Wellness Arm	0.74	0.58	0.94		0.78	0.48	1.25		0.21	−0.04	0.45	
By Pre-pregnancy BMI												
Overweight												
Exercise Arm	0.61	0.47	0.79	0.83	0.70	0.50	0.96	0.64	−0.04	−0.34	0.28	0.94
Health and Wellness Arm	0.57	0.43	0.75		0.53	0.31	0.93		0.18	−0.09	0.45	
Obese												
Exercise Arm	1.05	0.87	1.26	0.31	0.79	0.57	1.10	0.87	−0.12	−0.30	0.06	0.06
Health and Wellness Arm	0.82	0.65	1.02		0.86	0.65	1.13		−0.004	−0.19	0.18	

To determine if the intervention effects on CRP differed by ethnicity, we performed a stratified analysis among non-Hispanic and Hispanic women (Table 2). In non-Hispanic women, CRP declined to a greater degree in the exercise intervention arm (−0.26 mg/dL, 95 % CI: −0.55, 0.04) than in the health and wellness arm (−0.07 mg/dL, 95 % CI: −0.24, 0.11), although the difference in change in CRP only bordered on statistically significance (*p* = 0.08). In Hispanic women, CRP decreased slightly in the exercise arm (−0.04 mg/dL, 95 % CI: −0.23, 0.14) and increased in the health and wellness arm (0.21 mg/dL, 95 % CI: −0.04, 0.45); however differences in change in CRP between the arms was not statistically significant (*p* = 0.42).

We then repeated our analysis stratified by prepregnancy BMI as previous studies have shown differences in the impact of an exercise intervention on CRP according to obesity [29]. Among women that were obese at baseline, there was a larger decrease in CRP from baseline to post-intervention in the exercise arm relative to the health and wellness arm (exercise, −0.12 mg/dL, 95 % CI: −0.30, 0.06; health and wellness, −0.004 mg/dL, 95 % CI: −0.19, 0.18) (*p* = 0.06) that bordered on statistical significance (Table 2).

Next we performed a sensitivity analysis excluding women who were not compliant with the exercise intervention, which was defined as a self-reported decrease in time spent in sports/exercise of moderate intensity or greater from baseline to post-intervention (42.9 %) (Table 3). Similar to the findings in the overall sample, there was a non-significant decrease in CRP in the exercise arm (−0.10 mg/dL, 95 % CI: −0.29, 0.08) and increase in CRP in the health and wellness arem (0.08 mg/dL, 95 % CI: −0.07, 0.24). However, the difference in change in CRP between the intervention arms was not statistically significant (*p* = 0.11) (Table 3).

**Table 3:** Distribution of C-reactive protein at pre- and post-Intervention by Intervention arm excluding non-compliant participants from the exercise arm; The B.A.B.Y. Study, 2007-2012

	Pre-intervention				Post-intervention				Change			
	Geometric mean	95 %	CI	p-value	Geometric mean	95 %	CI	p-value	Mean (mg/dL)	95 %	CI	p-value*
Exercise Arm^a^	0.88	0.72	1.07	0.2	0.76	0.56	1.02	0.96	−0.10	−0.29	0.08	0.11
Health and Wellness Arm	0.68	0.57	0.82		0.69	0.51	0.92		0.08	−0.07	0.24	

*p-value for difference in change from baseline to post-intervention between groups

^a^Excluding women from the exercise arm that decreased/maintained time spent in sports/exercise from baseline to post-intervention

**Fig. 2:**
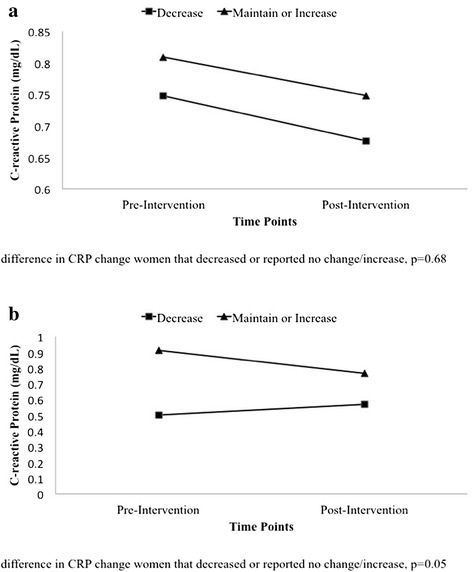
**a** Change in Mean C-Reactive Protein from Pre- to Post-Intervention according to Self-Reported Change in Moderate- to Vigorous-Intensity Activity; The B.A.B.Y. Study, 2007–2012. **b** Change in Mean C-Reactive Protein from Pre- to Post-Intervention according to Change in Sports/Exercise Activity; The B.A.B.Y. Study, 2007–2012

Finally, we performed a secondary analysis to examine the impact of self-reported change in physical activity on change in CRP. Women who decreased sports/exercise activities of moderate intensity or greater from baseline to post-intervention experienced a smaller decrease in CRP (−0.01 mg/dL, 95 % CI: −0.23, 0.21) than women who maintained/increased these activities (−0.05 mg/dL, 95 % CI: −0.16, 0.06). However, the differences in change in CRP were not statistically different between the two groups (*p* = 0.68) (Fig. 2a). In terms of sports/exercise, there was a small but statistically significant difference in change in CRP between women who decreased and women that maintained/increased their activity from baseline to post-intervention (*p* = 0.046). Specifically, women that decreased their time in sports/exercise experienced a mean increase in CRP (0.09 mg/dL, 95 % CI: −0.14, 0.33), whereas women who maintained or increased their sports/exercise experienced a mean decrease in CRP (−0.08 mg/dL, 95 % CI: −0.23, 0.08) (Fig. 2b).

## Discussion

Findings from this randomized trial of an individually-tailored motivationally-matched exercise intervention in an ethnically and socio-economically diverse population of pregnant women at high risk for GDM were consistent with a positive impact of the exercise intervention on CRP levels, but not of statistical significance. After the 12-week intervention, women in the exercise intervention experienced a 0.09 mg/dL (i.e., 3 %) decrease in CRP while the comparison health and wellness arm experienced a 0.08 mg/dL (i.e., 24 %) increase in CRP. Findings were virtually unchanged when restricting the analysis to women that were compliant with the exercise intervention protocol. Stratifying the analyses according to ethnicity or prepregnancy BMI suggested that the positive impact of the exercise intervention might be limited to women that were obese prepregnancy. Finally, there was a suggestion of a beneficial impact of increasing self-reported time spent in sports/exercise on CRP levels.

To our knowledge, this is the first study to examine the impact of an exercise intervention on CRP levels in a pregnant population. In non-pregnant populations, the impact of physical activity interventions on CRP has been conflicting. A review of 11 randomized trials that used either aerobic training or lifestyle (diet and exercise) interventions reported reductions in CRP ranging from 30 % to 53 % [13]. For example, a two-month weight reduction program by Okita et al. that involved 3 days/week of moderate intensity aerobic training (2 days supervised and 1 day at home) in 199 apparently healthy women resulted in a 0.022 mg/dL (i.e., 35 %) reduction in CRP [30]. A year-long moderate intensity aerobic exercise intervention in 320 postmenopausal women by Friedenreich et al. led to modest reductions in CRP. CRP reduced from 1.4 mg/dL to 1.1 mg/dL in women in the exercise arm, whereas no change was reported in the usual activity control group [31]. Similarly, Dvorakova-Lorenzova et al. reported a 0.13 mg/dL (i.e., 30 %) reduction in CRP after a 9-week diet and physical activity intervention that consisted of one hour of moderate intensity aerobic training 3 days/week in 40 young obese women [32]. By way of comparison, in the current study we observed a 0.09 mg/dL reduction in CRP in the exercise arm. However, our study relied predominantly on mail and telephone to deliver the intervention and did not include in-person aerobic training sessions, which may have attenuated its impact [33]. Additionally, the physiological response to exercise is likely different during pregnancy than in non-pregnancy.

Arikawa et al. randomized 319 apparently healthy women to either a 16 week supervised moderate intensity aerobic exercise intervention or a no exercise control group [29]. The exercise intervention led to reductions in CRP, which were largely driven by its effect in obese women. Specifically, in obese women, aerobic exercisers experienced a 4.38 mg/dL decrease in CRP compared a 1.44 mg/dL increase in CRP in non-exercisers (*p* = 0.03). Our findings were consistent with Arikawa’s, indicating a marginal impact of the intervention on CRP in obese women. However, our power to detect such an effect was likely limited by the fact that obese women were more likely to be missing CRP data than women who were not obese.

In contrast, other studies have found no impact of an exercise intervention on CRP levels among non-pregnant women. Hammet et al. conducted a 6 week (3 days/week) supervised moderate intensity aerobic training intervention in 152 female smokers [34]. At the end of the 6-week intervention, CRP was unchanged in both the exercise and controls groups. At the 12-week follow-up, there was a non-significant 0.01 mg/dL decrease in CRP in the exercise group and no change in CRP in the control group (*p* = 0.49). Campbell et al. reported similar null findings in a year-long moderate intensity aerobic intervention (45 min/day, 6 days/week; 3 supervised, 3 home based) in 202 men and women. In an aerobic exercise group, there was a non-significant 0.021-mg/dL decrease in CRP and no change in a stretching exercise group (*p* = 0.65) [35]. However, in a subset of obese post-menopausal women, there was a 0.079 mg/dL reduction in CRP in the aerobic exercise group compared to an increase in the stretching exercise group [36]. Reed et al. conducted a 4-month diet and exercise intervention on 24 healthy young women (~25 years of age) [37]. The exercise component consisted of 40–90 min of supervised moderate intensity aerobic training 4 days/week. Despite reporting a small increase in total energy expenditure, there was only a non-significant 0.05 mg/dL increase in CRP (*p* = 0.62).

The current manuscript extends the previous findings by examining the association between physical activity and CRP in an ethnically and socio-economically diverse population of pregnant women at high risk for GDM. Our analysis showed a decrease of 0.09 mg/dL after a 12-week exercise intervention. Whether this decrease in CRP represents a clinically meaningful reduction is less understood. Teran et al. compared variations in CRP over the course of pregnancy in women who subsequently developed preeclampsia (*n* = 24) to women who had an uncomplicated pregnancy (*n* = 183) [6]. CRP levels were similar between groups in early pregnancy, however, CRP levels were elevated in mid to late pregnancy in women who developed preeclampsia compared to women with who had uncomplicated pregnancy. Additionally, Qiu et al. examined the relationship between CRP in early pregnancy (~13 week gestation) and incidence of GDM in 855 women participating in the OMEGA study [38]. The authors found that every 0.1 mg/dL increase in CRP was associated with a 20 % increased risk of developing GDM. The effect sizes observed in this study were similar to the magnitude of decrease in CRP observed in the current manuscript. However, these finding must be interpreted with caution since the effects of change in CRP and risk for adverse maternal outcomes were not examined.

The mechanisms by which physical activity may impact CRP levels are poorly understood. Some reports suggest the relationship between physical activity and CRP is mediated through adiposity [39]. In pregnant populations, obesity is associated with elevated CRP levels [40], however, weight gain over the course of pregnancy is not associated with change in CRP. Further, in the current manuscript, the relationship between physical activity and CRP was independent of gestational weight gain. Physical activity may also impact CRP through its association with other pro- and anti-inflammatory cytokines. Specifically, physical activity has been associated with pro-inflammatory cytokines such as TNA-α and IL-6, which stimulate CRP production [41]. Additionally, physical activity has been associated with anti-inflammatory cytokines such as IL-1 and IL-10, which inhibit CRP production [41, 42]. Additional studies are needed to further elucidate the mechanisms of the association of physical activity and change in CRP during pregnancy.

While we observed only small changes in CRP associated with the intervention, there were limitations that may have attenuated our results. First, the main analysis used an intention-to-treat approach, which examines the effect of the intervention, but may mask the benefits of physical activity if participants in the intervention group were non-compliant with intervention protocols or if the exercise goals were too modest to have an effect on CRP. In our recently published paper evaluating the effect of the exercise intervention on change in activity in the B.A.B.Y. Study, the exercise intervention group increased their sports/exercise activity from a mean (SD) of 7.9 (11.2) to 13.1 (11.4) MET hours/week while the health and wellness group increased their sports/exercise from 6.7 (7.8) to 7.0 (9.1) MET hours/week. The difference in change between groups (5.3 vs. 0.3) was statistically significant, *p* = 0.002). In addition, the exercise group was more likely to achieve ACOG guidelines for physical activity as compared to the health and wellness group (odds ratio = 2.12; 95 % confidence interval = 1.45, 3.10) [43].

While our primary analysis was intent-to-treat, we conducted a secondary analysis categorizing women according to their self-reported change in physical activity over the course of the intervention. We found a small, but statistically significant difference in change in CRP between women who decreased and women that maintained/increased their activity from baseline to post-intervention. One limitation of this analysis is the lack of an objective measure of physical activity, which may have yielded a stronger association. Self-reported measures have been shown to be more weakly associated with biomarkers than objectives measures [8]. Further, self-reported measures could be affected by problems with recall and social desirability. However, the PPAQ has been shown to be both a valid and reliable measure of physical activity in this sample population [27]. In addition, physical activity was assessed prospectively and women were not aware of their CRP levels at the time of their reporting. Another limitation was the lack of information on the potential confounding factors of pre-pregnancy physical activity, dietary intake, and blood volume. However, as the B.A.B.Y. study was a randomized trial, it would be anticipated that these factors would be balanced between study arms. Indeed, the baseline distribution of the majority of socio-demographic, medical history, and behavioral variables did not differ between study arms indicating that the randomization was successful. Finally, while we had 80 % power to detect a clinically significant mean difference in change in CRP of 0.76 mg/dL, our observed findings were not statistically significant likely due to insufficient power to detect the smaller range of observed differences of slightly less than 0.1 mg/dL.

## Conclusions

In conclusion, a 12-week exercise intervention did not result in a statistically significant impact on CRP in pregnant women at high risk for GDM. However, our observation of a suggestion of a decrease in CRP levels is consistent with the findings of prior observational and experimental studies. These findings extend this prior literature by being the first to examine the impact of physical activity on CRP levels in pregnant women. Additional studies are needed to better elucidate the relationship between change in physical activity and CRP in pregnant women.
